# Beyond the ceremony: Mega-event, air quality and political career

**DOI:** 10.1371/journal.pone.0262470

**Published:** 2022-02-02

**Authors:** Li Fang, Pan He, Chuanhao Tian, Yao Yao, Hongjie Chen

**Affiliations:** 1 Departmen of Urban and Regional Planning, Florida State University, Tallahassee, Florida, United States of America; 2 School of Earth and Ocean Sciences, Cardiff University, Cardiff, Wales, United Kindom; 3 School of Public Affairs, Zhejiang University, Hangzhou, Zhejiang, China; 4 Center of Social Welfare and Governance, Zhejiang University, Hangzhou, Zhejiang, China; Public Library of Science, UNITED STATES

## Abstract

This paper examines whether mega-events-initiated planning regulations improved air quality in Chinese cities and explores the driving forces of the enforcement of such regulations. Using the 2008 Beijing Olympics as an example, we find that mega-events serve as an opportunity for cities to combat air pollution. The 2008 Olympics prompted a handful of Chinese cities to cut pollution and sustain a blue sky: Cities with air quality regulated for the Olympics cut their Air Pollution Index by about 16 points during the Games, compared to non-regulated cities, and 60% of that effect remained four years after the event. These achievements are obtained through effective mobilization of city leaders by associating air quality with their political careers. This study reveals that 1) a mega-event may improve urban environmental quality beyond the host cities and the event period, and 2) successful implementation of environmental regulations hinges on incentivizing local leaders.

## Introduction

Urban air pollution in China has become a severe issue. Among 20 world’s most polluted cities, sixteen were found in China in 2007 [1]. Particulate concentrations in most Chinese cities were far above the World Health Organization Air Quality Guidelines [2]. In early 2013, the heavy smog and haze in Beijing pushed the air pollution index to a record high [3], and the concentration of PM2.5 was beyond index–higher than the maximum 755 μg the US Embassy’s equipment can measure [4]. Severe air pollution threatens public health [5] and leads to substantial economic and welfare losses [6, 7]. 450,000 deaths in China were attributed to the PM2.5 emission in 2000 [8].

China’s air pollution crisis affects the globe. Jia and Ku (2017) found that China’s heavy smog chocked South Korea, its immediate Eastern neighbor [9]. Studies also suggested that China’s air pollution traveled all the way to the west coast of the U.S. [10] These make the issue of China’s smog a global concern.

To curb the air quality from further deterioration, Chinese governments have taken on the fight. The central government launched the Two Control Zones policy in 1998 to reduce SO_2_ and acid rain in most polluted cities. Also, starting from the 11th Five-Year Plan (2006–2010), local governments are held responsible for specific emission targets, with the enforcement linked to local leaders’ career prospects. These measures were effective [11, 12]: Five years after the Plan, SO_2_ abatement targets were met with a 14.29% reduction in SO_2_ emission [12].

Nevertheless, China’s trip from smog to blue sky is still bumpy. China’s subnational politicians are ready to pollute their jurisdictions in exchange for impressive economic growth [13]. This is because local politicians’ career outlook largely depends on local economic performance [14–17]. The reversal of this regime requires prioritizing environmental quality over economic growth. This is no small issue and may take a long time to achieve.

Mega-events may accelerate the change [18]. The pressing demand for a clear sky to accommodate worldwide attentive events pushes governments for changes. In recent years, China has established a reputation as a capable host of mega-events and was widely recognized for securing a spotless sky during these ceremonies [19]. The blue sky is sustained through both temporary command-and-control measures and permanent regulations. Examples of the former include plant suspension and vehicle restrictions, and those of the latter consist of stricter emission standards and the inclusion of emission targets into politicians’ performance evaluations. While the effect of the former may be transient, that of the latter persists [20].

This paper quantifies the extent to which environmental regulations initiated for mega-events can improve air quality, and more importantly, whether such an effect persists after the events. Specifically, we have examined (1) the magnitude of the pollution cut during the mega-event, (2) the persistency of such effect after the event, and (3) the mechanism through which the regulations are successfully enforced. This paper uses the 2008 Beijing Olympics as an example of mega-events because it involves multiple host cities and an extended event period. Most mega-events, such as the Asia-Pacific Economic Cooperation (APEC) CEO Summit and the G20 Summit, are hosted in a single city, last shorter than a week, and have fewer than five neighboring cities regulated for air pollution. These features add to the statistical difficulty of detecting the changes in air quality. The 2008 Olympics perfectly solves this problem. It has six host cities in mainland China and over 30 neighbors regulated for air pollution. In addition, the game lasts for 19 days. Thus, it is much more feasible to conduct statistical tests on the change in air quality.

The contribution of this paper is four-fold. First, following Chen et al. (2013) [20], it quantifies the effect of the Olympics on air quality in Chinese cities. While Chen et al. (2013) focus on Beijing and its neighbors [20], this paper expands to a much wider range of cities regulated for the Olympics. It also explores the mechanism behind the effect–why these air pollution regulations are effectively enforced at the local level while many others have failed. Second, it adds to the literature on “green” governance [21]. It shows that a nation may seize the opportunity of mega-events to improve environmental planning. Third, this paper adds to the literature on the impacts of mega-events [22], and shows that mega-events exhibit a positive effect on environmental quality [23, 24]. Uniquely, this paper shows that these impacts expand beyond the host cities.

## Literature

### Mega-events, the 2008 Olympics and the concerns for air quality

Mega-events carry significant political meanings: they signal economic and technological achievements [25], attract international media coverage [22], overcome a historic (usually negative) image and place the host country more centrally within the global community [26]. These benefits are appealing to many countries, and certainly to China.

Thus, as the first summer Olympics that China has ever hosted, the 2008 Beijing Olympic Games means much more than a sport event. It was loaded with tremendous political significance, and the preparatory work was given high priority and abundant resources. About RMB 290 billion ($42 billion) was spent on infrastructure, sports facilities, environmental protection equipment (RMB 71.3 billion), operating expenses and security and others. The magnitude of the expenditure trumps the second most expensive Olympics, the 2004 Athens Games, by 2.5 times, and accounts for 43% of all expenditures for modern Olympics since 1976 [27].

**Fig 1:**
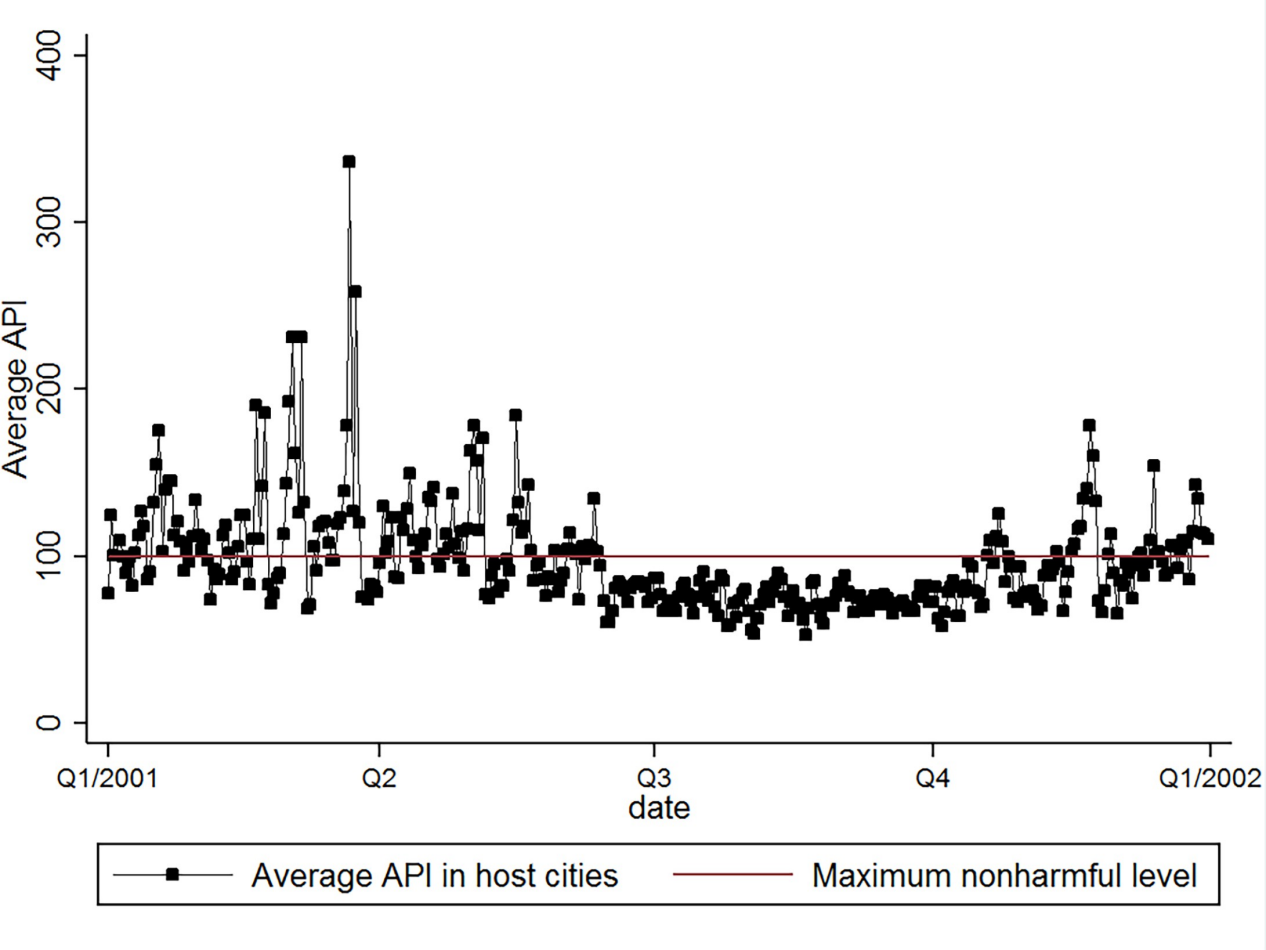
Average Air Pollution Index (API) in host cities in 2001. The maximum nonharmful level is defined by the State Environmental Protection Administration of China.

Despite China’s promise of a spectacular and impressive pageant, a number of concerns surfaced. One of them is the air quality and its potential health effects on the athletes. In 2001 when China succeeded in the Olympics bid, the host cities suffered from an air pollution level harmful for human health in over one-third of the days (Fig 1). Foreign media frequently covered the worries of the health effects, which led some athletes to wear masks, switch to competitions easier for lungs, or even withdraw from the event [28, 29].

### Olympic-relevant regulations on air pollution

Given the political implication of the game and the serious concerns of air pollution, the central government endeavored to cut pollution [30]. Beijing Organizing Committee for the Olympic Games took on air quality improvement in host cities as one of its core tasks and devoted RMB 140 billion ($20.43 billion) to it. A series of technical and administrative measures were implemented: Desulfuration, dust removal, and denitrification facilities were installed for firms including Yanshan Petrochemical, Beijing Thermal Power Plant and the power plant of Capital Steel. Pollution-intensive firms such as Capital Steel, Beijing Coking Plant, and Beijing Dyeing Plant were either shut down or relocated. Vehicle emission was also controlled, with stricter emission standards employed on March 1, 2008. Similar measures were adopted in co-host and neighbor cities. For instance, Shenyang invested 163 million RMB to replace old buses, and Shanghai installed desulfuration facilities for large electricity generating plants [20].

Besides these conventional measures, the central government also formulated a set of temporary command-and-control measures. The national authority of environmental regulation, the Ministry of Environmental Protection, formulated *The Assurance Measures of Air Quality during the 29th Olympic Games in Beijing*, followed by local governments with detailed plans. According to these documents, the six host cities in mainland China and over 30 neighbor cities were required to take actions from a few months before the game to the end of the event. For instance, approximately 150, 40, 300 and 132 polluting factories in Beijing, Tianjin, Tangshan and Shandong were ordered to suspend or reduce operations since early July, respectively [31, 32]. Beijing and Tianjin adopted an odd-even license plate rule during the event, which cut the traffic by half, i.e. more than a million automobiles [33]. Plus, the outdoor construction sites in multiple cities were halted [34]. The impact of these temporary command-and-control measures may be transient, but that of the more permanent technical and administrative measures could last beyond the Games period. Measures adopted in selected host and neighboring cities are summarized in S1 Table.

Aside from the six host cities in mainland China, Hong Kong is another host city for the 2008 Olympics. However, these air pollution regulations do not apply there due to Hong Kong’s high degree of autonomy. As a result, before the 2008 Olympics, no specific regulations have been issued for Hong Kong and its neighboring cities. Thus, these cities are excluded from this analysis.

These regulations worked well in sustaining air quality during the game period. Throughout the game, Air Pollution Index (API) remained under 100, the maximum non-harmful level, in Beijing; all game dates were graded “good” according to the benchmarks of API, and about half were “excellent.” Wang et al. (2009a) and Wang et al. (2009b) also suggested a significant reduction of black carbon and particle concentration during the periods of traffic control than otherwise [35, 36]. Chen et al. (2013) showed that these measures improved API in Beijing during and a little after the game, although a significant proportion of the effect faded away by October 2009 [20]. Such change also brings public health benefits, such as the reduction in acute respiratory inflammation in school children [37], asthma visits in adults [38] and all-cause mortality rate [39]. As a result, health-relevant costs declined by 38% and 16% compared with the pre- and post-game periods respectively [40].

### Urban environmental quality and political career

In China, political careers of subnational politicians hinge on a set of evaluation criteria established by their superiors. At the prefecture level, city leaders are evaluated, promoted and demoted by provincial party committees with 10–15 key provincial politicians. The career outcomes of prefecture city leaders depend on criteria such as economic performance (e.g., GDP and revenue growth rates), the completion of major political tasks (e.g., performance during mega-events), and more recently, environmental achievements (e.g., the abatement of sulfur dioxide).

In recent years, a pleasant environment has started to play a role in the evaluation of city officials. For instance, Zheng et al. (2014) found that the improvement of air quality enhances the probability of promotion for mayors [11]. A pleasant environment is also a prerequisite for a city to successfully accommodate mega-events. Thus, the importance of environmental quality during mega-events in relevant cities (e.g., host and neighbor cities) is double-loaded: It is not only by itself a benchmark for official evaluation, but also an essential element in the completion of a political task. This elevated importance should have motivated city leaders to secure a wonderful environment for mega-events, the idea of which is formally tested in this paper.

## Methods

Previous literature often adopts a before-after comparison to analyze the consequences of air pollution regulations during mega-events. For example, Chen et al. (2013) compare the air quality before, during and after the game to inspect whether the campaign against pollution for the 2008 Olympic Games works well [20]. Plausible as it seems, this cannot rule out the contemporary confounding factors.

In this study, we deal with this issue with a difference-in-difference method. Concerning the regulations, there are four types of cities: (1) host cities, which are all required to control air pollution for the games, (2) non-host cities that are required to control their pollution because of their closeness to the host cities, (3) non-host cities exempt from the campaign of pollution control despite their closeness to the host cities, and (4) other cities. Moreover, as mentioned previously, Hong Kong and its neighboring cities are not similarly regulated for the Olympics. Thus, Hong Kong is excluded from group (1) and its neighboring cities are classified into group (4).

**Fig 2:**
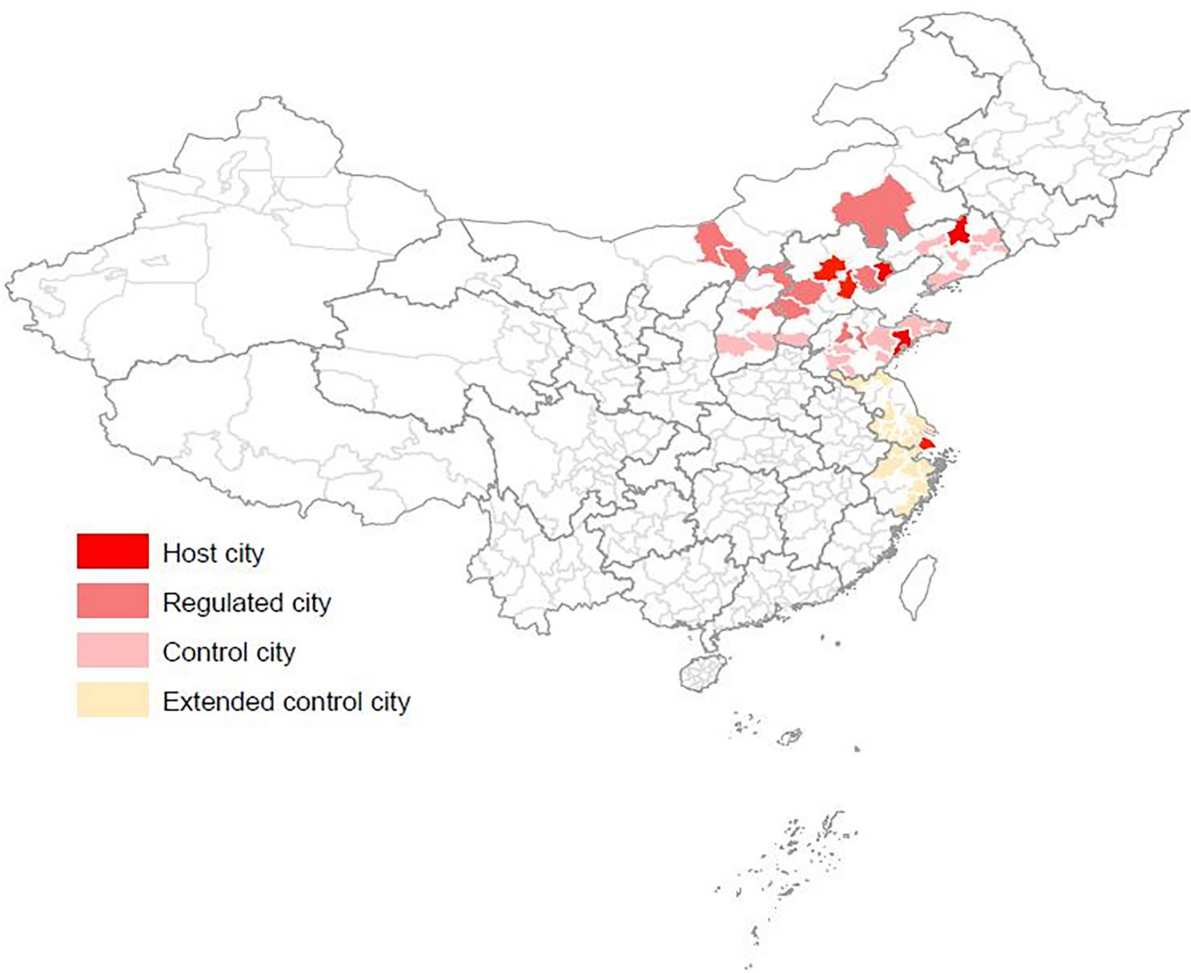
Geographical distributions of host cities, regulated cities and control cities.

We drop the host cities from the study because their preparation for the Olympics is a comprehensive package, and their preparative work in other fields may indirectly affect air quality and thus confound the effect of the air pollution regulations. For example, Beijing has largely expanded its open space for the Olympics, which surely uplifts air quality. Instead, we choose cities in group (2) as the treatment group. These cities are held responsible for air pollution reduction for the Olympics and no more. Thus, their improvement in air quality during the regulated and the game period can be attributed to the air pollution control regulations. We select cities in group (3) as the control group. These cities are not regulated for the Olympics, but locate in the same provinces as the regulated cities. They thus are spatially close to the host and the regulated cities. Using them as the control group eliminates most cross-provincial differences, and ensures the comparability of air quality and political career outcome between the treatment and control groups. Locations of treatment and control cities are shown in Fig 2.

Our study time frame is 2006 to 2012. To detect the effect of the regulations, air quality in the treatment and control groups is compared during four periods: The first period is before the Olympics regulations, which starts from January 1^st^, 2006 to the start date of regulation in each city. These regulations start on different dates in different cities, with the earliest date being November 7^th^, 2007. The second period is after the regulations but before the Games, which spans from the start date of the regulation in each city to August 5^th^, 2008. The third period is during the Games, from August 6^th^ to 24^th^ in 2008. And the final phase is after the Games, which starts from August 25^th^, 2008 and lasts till December 31^th^, 2012, the end of our study period. This design allows air quality to be valued differently by periods.

Four effects may confound our estimation. First, there can be a systematic difference in air quality between the treatment and control groups. The difference-in-difference method requires the treatment and control groups to share parallel trend in air quality before regulation, which we tested and found a parallel trend. Another possible confounding effect is the 11^th^ Five-Year Environmental Plan initiated in 2006. As mentioned above, this plan holds local officials responsible for meeting emission targets, which may have prompted cities to cut pollution. Nevertheless, as the start date of our study is 2006, the estimated effects can be interpreted as additional air pollution cuts induced by the Olympics. A third confounding effect comes from the seasonality of air quality. Since the Olympics was held in summer and the regulations came into force during winter and spring, the seasonality of air quality may affect the result. We control for date, month or month by climate district fixed effects to eliminate this concern. Last, there can be an anticipation effect–regulated cities anticipated that they were going to be regulated, so the local officials started to adapt earlier than the regulations kick in. We examine this later in a month-by-month analysis.

We estimate the effect of the air pollution regulations with Eq (1).

$$\begin{array}{l} {\mathrm{Air}}_{it}=\alpha_{0}+\alpha_{1}{\mathrm{rdate}}_{it}+\alpha_{2}{\mathrm{gdate}}_{t}+\alpha_{3}{\mathrm{pdate}}_{t}+\alpha_{4}{\mathrm{reg}}_{i}*{\mathrm{rdate}}_{it}+\alpha_{5}{\mathrm{reg}}_{i}*\\ {\mathrm{gdate}}_{t}+\alpha_{6}{\mathrm{reg}}_{i}*{\mathrm{pdate}}_{t}+\alpha_{7}X_{it}+a_{i}+yr_{t}+\varepsilon_{it} \end{array}$$ (1)

where *Air*_*it*_ denotes air quality in city *i* date *t*. *reg*_*i*_ is a dummy indicating that city *i* is regulated for air pollution for the Olympics. *rdate*_*it*_ denotes regulated dates for city *i*, lasting from the start of the air pollution regulations till the end of the Games on August 24^th^, 2008. *gdate*_*t*_ denotes Games dates from August 6^th^ to 24^th^ in 2008, and *pdate*_*t*_ denotes post-game dates, which starts on August 25^th^, 2008 and lasts till December 31^th^, 2012—the end of our study period. Regarding the pre-regulation phase as the baseline, we are interested in *α*_4_ to *α*_6_; each of them should be negative if the regulations result in a better air quality in regulated cities in the corresponding period compared to non-regulated cities. ***X***_***it***_ denotes a set of exogenous variables for city *i* on date *t* that also affect air quality, primarily climate factors. In accordance with Chen et al. (2013) [20], we control temperature, precipitation, wind, humidity and air pressure. These factors can affect the air quality through either pollution generation (e.g. cold weather raises pollution by heating) or diffusion (e.g. wind lowers pollution level by spreading out pollutants). *a*_*i*_ denotes city fixed effects. *y*_*t*_ denotes year fixed effects. *ε*_*it*_ denotes random errors. In alternative specifications, we also include date, month, or month by climate district fixed effects and to capture the seasonality of air quality. Finally, in an alternative specification without date fixed effects, we add a dummy variable for holidays to capture the difference in emission between work and non-work days.

Next, we explore whether the career incentive of city officials explains the enforcement of the regulations. Our analysis focuses on both mayors and China’s Communist Party (CCP) secretaries. In China, two lines of officials govern cities: party officials and executive officials. Party officials, led by the CCP secretary, hold the power to major public affairs. Executive officials, led by the mayor, are responsible for day-to-day executive issues. Previous studies tend to focus on mayors when examining the association between environmental quality and political promotion [11, 12, 41], arguing that mayors are in charge of environmental affairs at the city level. However, it is the CCP secretary—the local official with the highest administrative ranking—that takes the ultimate responsibility for top-down political tasks [12]. Therefore, it is unlikely that secretaries are dissociated from the implementation of air pollution regulations for mega-events.

We measure an official’s career outcome by whether he or she is promoted or demoted. A city official is considered promoted if he or she: 1) joins the provincial CCP standing committee, 2) becomes a principal of a provincial-level position, 3) becomes a deputy of a provincial party committee, government, people’s congress or people’s political consultative committee, 4) joins the central government, 5) moves from an prefectural city to a sub-provincial city, while keeping the same position level, or 6) gets a higher-ranking position in the same city (only for mayors, as the CCP secretary is already the highest ranking position in a city). On the other hand, an official is considered demoted if he or she: 1) moves to lower-ranking positions in the same city or same-level cities, 2) relocates to a lower-ranking city while keeping the same position level, or 3) becomes a deputy of a provincial-level position outside of the provincial party committee, government, people’s congress and people’s political consultative committee. We exclude officials with abnormal changes, including death, serious illness, investigation for corruption, and physical disappearance.

We control for economic performance of a city, as it stands out as a major determinant of local politicians’ career. For example, Yao and Zhang (2011) found that GDP and revenue growth rates uplift the probability of promotion of city leaders [42]. In this paper, we include annual growth rates of GDP and fiscal revenue, in accordance with Yang and Zheng (2013) [43] and Chen et al. (2017) [44].

We also control for individual characteristics to mitigate the concern that the political career outcome is affected by the inherent abilities or political networks of individuals other than their performance on the current position [41, 43, 45, 46]. We include demographic variables, past education experience and work trajectory, and political network. Demographic control variables include age in quadratic form, sex, and ethnic group. We control for education experience with dummy variables of education levels, academic disciplines, and studying experience abroad. We also control for a rich set of variables related to work experiences, including service term (in month) in quadratic form, years of work, years as a CCP member, whether a native, and experiences in specific institutions—whether the official has worked in the central government, provincial governments, universities or research institutions, state-owned enterprises, China Communist Youth League, the organizational departments, oversea institutions, the economic sector, and as a secretary in previous terms, following the literature [11, 41, 42, 44, 46, 47]. Finally, we include an index to capture the social network of a city politician j, measured by the percentage of provincial CCP standing committee members that share the same hometown or the same university with j, following Opper, Nee and Brehm (2015) [46]. Opper, Nee and Brehm (2015) argued that politicians originated from the same city or went to the same school form a relatively strong social network and would look after each other in their political endeavors [46].

Putting these together, we test how the career of city officials is affected by air quality preceding and during the Olympics with a difference-in-difference-in-difference analysis as follows.

$$\begin{array}{l} y_{ijt}=\gamma_{0}+\gamma_{1}{\mathrm{Air}}_{it}*{\mathrm{reg}}_{i}*{\mathrm{rdate}}_{it}+\gamma_{2}{\mathrm{Air}}_{it}*{\mathrm{reg}}_{i}*{\mathrm{gdate}}_{t}+\gamma_{3}{\mathrm{Air}}_{it}*{\mathrm{reg}}_{i}*\\ {\mathrm{pdate}}_{t}+\gamma_{4}{\mathrm{Air}}_{it}+\gamma_{5}{\mathrm{Air}}_{it}*{\mathrm{rdate}}_{it}+\gamma_{6}{\mathrm{Air}}_{it}*{\mathrm{gdate}}_{t}+\gamma_{7}{\mathrm{Air}}_{it}*{\mathrm{pdate}}_{it}+\gamma_{8}{\mathrm{reg}}_{i}*\\ {\mathrm{rdate}}_{it}+\gamma_{9}{\mathrm{reg}}_{i}*{\mathrm{gdate}}_{t}+\gamma_{10}{\mathrm{reg}}_{i}*{\mathrm{pdate}}_{t}+\gamma_{11}{\mathrm{Air}}_{it}*{\mathrm{reg}}_{i}+\gamma_{12}{\mathrm{rdate}}_{it}+\\ \gamma_{13}g{\mathrm{date}}_{t}+\gamma_{14}pdate_{t}+\gamma_{15}W_{it}+\gamma_{16}Z_{ijt}+yr_{t}+a_{i}+\varepsilon_{ijt} \end{array}$$ (2)

where *y*_*ij*,*t*_ denotes the career outcome for politician *j* in city *i*, when *j*’s service term covering date *t* ends. Note that the service term may not end at date *t*, but last a few more years after *t*. We assume that an official’s performance, including air quality performance, over the entire service term covering date *t*, can affect the probabilities of promotion and demotion at the end of the term. We separately study the probabilities of promotion and demotion to allow for asymmetric career incentives. In the promotion regression, *y*_*ijt*_ = 1 denotes promotion and 0 denotes demotion, retainment or lateral move; in the demotion regression, *y*_*ijt*_ = 1 indicates demotion and 0 indicates other outcomes. ***W***_***it***_ is a set of city economic performance variables. ***Z***_***ijt***_ is a set of politician characteristics. Other variables are the same as in Eq (1), which include year and city fixed effects. These two sets of fixed effects control for the waves of promotion/demotion in election years and how promising a city is for politicians. *γ*_1_ and *γ*_2_ are the coefficients of interest, and should be negative (positive) if air quality in regulated cities during the critical periods is more tightly connected to the promotion (demotion) of city officials. These capture the additional connection between air quality and officials’ career outcomes during these critical periods, when securing high air quality is a political task. Outside of these critical periods, air quality after 2006 is also an evaluation criterion for officials’ promotion, the importance of which is captured by *γ*_4_.

## Materials

Our data come from multiple sources. Air quality is measured by API, a comprehensive indicator of the level of air pollution. It is promulgated by the Ministry of Environmental Protection from 2001 to communicate air quality and alert the public potential air-related health risk in an intuitive way. This indicator evaluates air pollution level based on concentrations of five major pollutants: particulate matter (measured in particles less than or equal to 10 micromemters in diameter, PM10), nitrogen dioxide (NO_2_), sulfur dioxide (SO_2_), carbon monoxide (CO), and ozone (O_3_). APIs are calculated daily at the city level. With pollutant concentrations from each monitoring station within a city, local Environmental Protection Bureaus takes their daily average, and then translates the records into API. API ranges from 0 to 500, with higher value indicating more severe pollution.

Individual characteristics of city officials are collected from multiple online sources including officials’ resumes, websites of reputable news media, and official websites of local governments. We have built this database in the past decade, and made it one of the city official databases that contain the most comprehensive individual characteristics. Variables related to this study are made publicly available via GitHub. Socio-economic characteristics of cities including annual growth rates of GDP and fiscal revenue come from China City Statistical Yearbooks.

Climate variables come from China Meteorological Data Sharing Service System. This system contains daily meteorological records from more than 800 county-level weather stations including temperature, precipitation, wind, humidity, etc. We geocode all the stations and relate them to cities in our sample. For cities with more than one station, the average of these stations is adopted, while cities with no station are related to the nearest station.

We focus the analysis on the period of 2006 to 2012. This period covers the entire duration of the 2008 Olympics and the implementation of game-related air pollution regulations. In addition, this study period spans over sufficiently long periods before and after the event; the former serves as the baseline while the latter sheds light on the non-transient effect of mega-events. Moreover, during the study period, API consistently served as the main indicator of air quality in China and the method of calculation kept the same, while after 2012, the index experienced significant changes.

## Results

### Descriptive statistics

**Fig 3:**
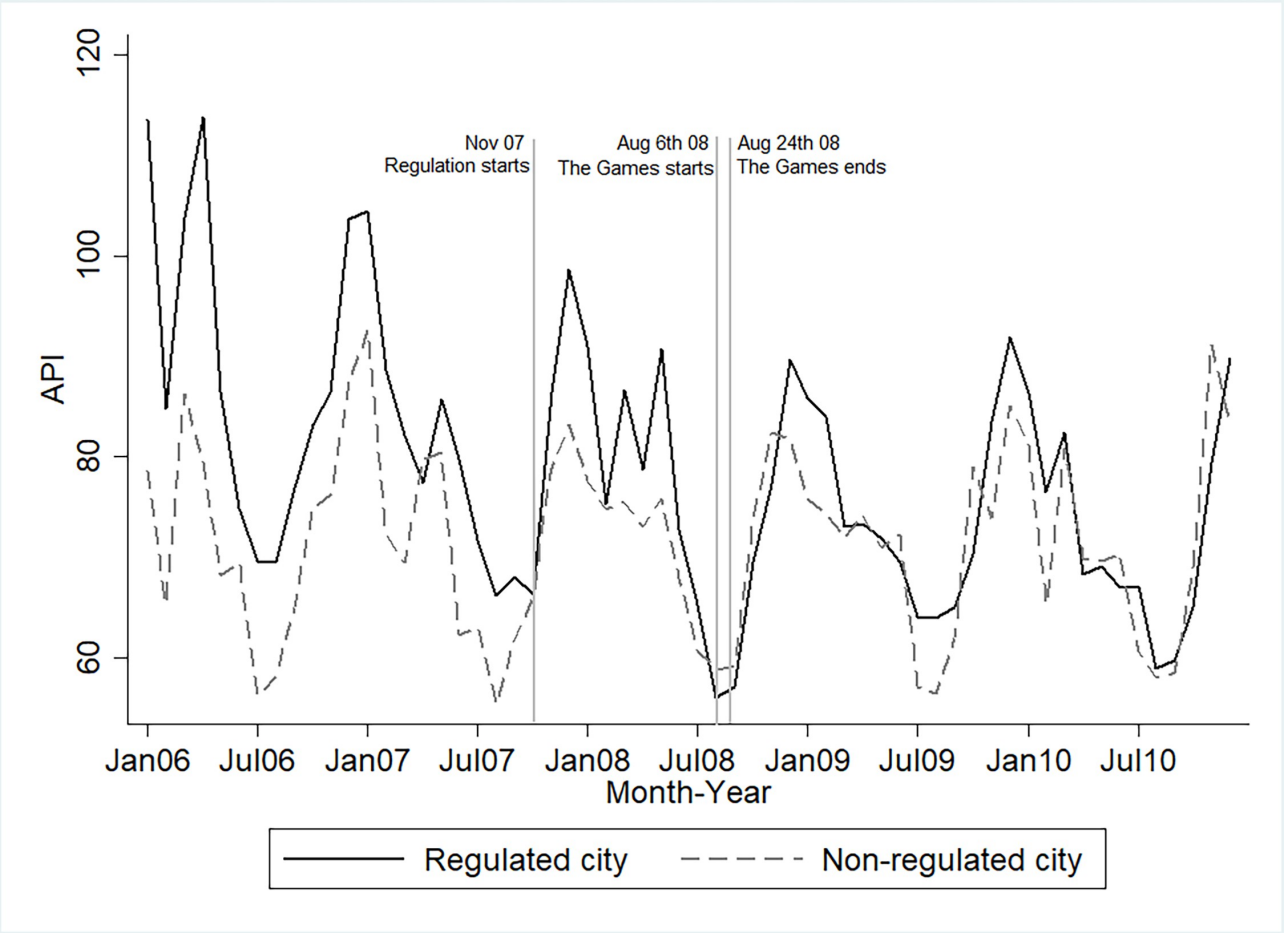
Average month-to-month API in regulated and non-regulated cities. November 8th, 2007 is the earliest start date of the regulation among all cities. On that date, Hebei province initiated an air quality regulation on six cities. Regulations on other cities gradually phased in after that, with the start date varying from November 2007 to July 2008.

Fig 3 contrasts month-to-month average APIs in regulated and non-regulated cities during different periods. Prior to the air pollution regulations, regulated cities were more polluted than non-regulated cities. In addition, these two types of cities, in general, share a parallel trend in their average APIs in the pre-regulation period, with an exception in April 2007.

After the phase-in of the Olympic-related regulations in November 2007, regulated cities immediate cut pollutions. However, their pollution level rebounded by the end of 2007. Nonetheless, their continued efforts to improve air quality are testified by their similar level of APIs with non-regulated cities after April, 2008. During the Games dates, regulated cities consistently outperformed non-regulated cities with lower APIs. After the event, air pollution levels in both groups climbed back, while in the relative term, regulated cities have kept similar air pollution levels with non-regulated cities, at least till the end of 2010.

Table 1, using descriptive analysis, compares career outcomes of CCP secretaries in regulated and non-regulated cities with different air pollution levels, from the prior-regulation period to the post-game period. Before the regulations, CCP secretaries in regulated cities with above median API (more polluted) were associated with an approximately 30% greater probability of promotion than those with below median API. But after the regulations, things turned around: Compared with those in the prior-regulation period, chances of promotion for CCP secretaries in polluted regulated cities became smaller by about 31% between the start of the regulations and the games, 34% during the games, and about 10% after the game. The chance of demotion shows a similar pattern. After the regulations, CCP secretaries in dirtier regulated cities were associated with a greater chance of demotion in each of the later periods, but after the Games, the chance of demotion in dirtier cities decreased. These results indicate that higher-level governments may have used both promotion and demotion as incentives to induce compliance among CCP secretaries in regulated cities. In contrast, political career in non-regulated cities tells a different story. CCP secretaries in non-regulated cities with worse air quality are always more inclined to promotion. They were also more inclined to demotion during regulated and the Games periods, but much less so compared to their counterparts in the regulated cities. Besides the different incentives laid out for regulations, CCP secretaries in regulated cities are in general less prone to promotion but more to demotion. This reveals the importance of city fixed effects in Eq (2) as politicians in different cities appear to have different career trajectories.

**Table 1:** Descriptive statistics of promotion and demotion of CCP secretaries in cities with above and below median API in different phases.

Period	Probability of promotion		Probability of demotion	
	API below median	API above median	API below median	API above median
*Panel A*: *regulated cities*				
Before regulation	3.74%	33.61%	10.50%	41.15%
During regulation but before game	3.82%	2.76%	5.45%	67.78%
During game	33.33%	0%	0%	66.67%
After game	24.13%	23.25%	15.72%	37.52%
*Panel B*: *non-regulated cities*				
Before regulation	44.34%	82.92%	8.17%	0%
During regulation but before game	42.27%	50.10%	0%	17.03%
During game	44.44%	45.45%	0%	11.11%
After game	25.54%	41.67%	13.63%	9.02%

Note: Cities with API above median are more polluted.

Table 2 shows the descriptive results for mayors. Mayors in more polluted regulated cities faced a greater change of promotion before regulation, but afterwards, their chance of promotion became thinner than those in the cleaner regulated cities. At the same time, their chance of demotion is always greater than those in the cleaner cities; and the difference inflated between the start of the regulations and the end of the Olympics. In contrast, among non-regulated cities, heavier polluters are less inclined to promotion and more inclined to demotion before regulation. However, they are always associated with a greater chance of promotion and a smaller chance of demotion after regulation.

**Table 2:** Descriptive statistics of promotion and demotion of mayors in cities with above and below median API in different phases.

Period	Probability of promotion		Probability of demotion	
	API below median	API above median	API below median	API above median
*Panel A*: *regulated cities*				
Before regulation	61.39%	68.95%	13.13%	31.05%
During regulation but before game	75.47%	64.20%	0%	35.80%
During game	75%	66.67%	0%	33.33%
After game	56.05%	48.79%	0%	0%
*Panel B*: *non-regulated cities*				
Before regulation	87.24%	81.96%	0%	9.43%
During regulation but before game	60.44%	88.62%	14.75%	0%
During game	45.45%	90%	18.18%	0%
After game	53.05%	62.63%	11.84%	8.07%

Note: Cities with API above median are more polluted.

### Effect of the air pollution regulations

Table 3 shows the estimation results for Eq (1), with descriptive statistics in S2 Table. Column (1) in Table 3 includes only the regulation status, different phases and city and year fixed effects. API decreased by 3.5 points in regulated cities compared to that in non-regulated cities after the regulation. The difference peaked during the Games dates with an additional API reduction of 11 points in regulated cities–a total of 14.5 points reduction. After the Games, this difference persisted, but the magnitude shrunk to 11.6 points.

**Table 3:** Effect of the air pollution regulations and the Olympics.

	API				
	(1)	(2)	(3)	(4)	(5)
Reg*rdate	‒3.512***	‒3.491*	‒4.564**	‒4.082***	‒5.246***
	(1.120)	(1.995)	(1.974)	(1.105)	(0.996)
Reg*gdate	‒11.181***	‒13.229***	‒12.399***	‒12.801***	‒10.453***
	(2.081)	(3.791)	(4.099)	(4.152)	(3.048)
Reg*pdate	‒11.619***	‒9.568*	‒9.791*	‒9.695*	‒9.841***
	(5.355)	(5.668)	(5.590)	(5.788)	(0.573)
X	N	Y	Y	Y	Y
City	Y	Y	Y	Y	Y
Year	Y	Y	Y	Y	Y
Holiday	N	N	Y	N	N
Month	N	N	Y	N	N
Date	N	N	N	Y	Y
Month* Climate district	N	N	N	N	Y
R^2^	0.0241	0.0941	0.1556	0.4805	0.2376
Number of Observations	50,973	42,459	42,459	42,459	42,459

NOTE: Standard errors clustered by city-year reported in the parentheses.

* p<0.1

** p<0.05

*** p<0.01.

These effects fluctuate a little as we control more city-level characteristics and include fixed effects for seasonality in columns (2) to (5). Column (2) controls for climate conditions. Column (3) adds month fixed effects to control for seasonality of air quality. A holiday dummy is also included to account for the difference in emission between work and non-work days. Column (4) includes date fixed effects to control seasonality at a more refined level. Finally, column (5) adds month by climate district fixed effects, to allow for different seasonal effects across climate districts. Specifically, the effect in regulated cities during regulated dates tends to increase. The additional effect during the Games dates is estimated between 10.5 to 13.2 points. The post-Games effect becomes smaller, between 9.6 to 9.8 points.

These results are qualitative the same with Chen et al. (2013) [20], but the coefficients are smaller. This can be reconciled if regulated cities around Beijing cut more pollution than other regulated cities, as Chen et al. (2013) focuses only on three cities neighboring Beijing [20]. Indeed, when we group the regulated cities into Beijing-neighbors and non-Beijing-neighbors, we find a significantly greater pollution alleviation effect in the former and the magnitudes are almost identical to those in Chen et al. (2013) [20].

**Fig 4:**
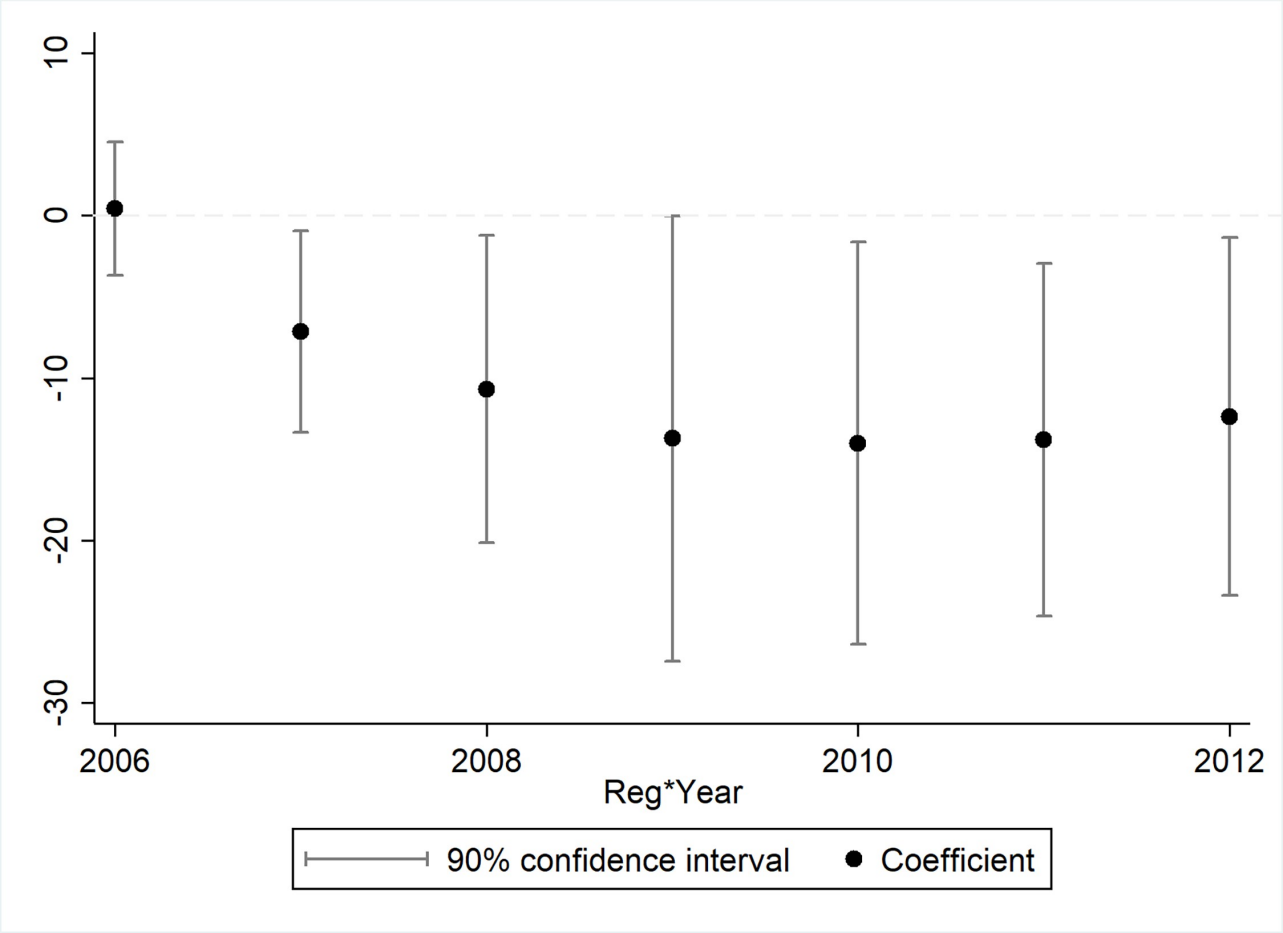
The coefficients of the year-by-year analysis of the different trend in API across regulated and non-regulated cities.

We have also tried other ways of dealing with time trends. The results remain robust when we add a flexible time trend polynomial up to the order of five (results not shown), following Fu and Gu (2017) [48]. A month-by-month analysis finds that the air pollution regulations took three months before they showed significant effects, after which they reduced API in regulated cities by 2 to 12 points. This exercise excludes the possibility of information leakage. Finally, a year-by-year analysis shows that the regulation effect started to emerge in 2007 and persisted till 2012 (results shown in Fig 4).

Moreover, the improved air quality implies significant economic and health benefits. For example, Matus, Nam, Selin, Lamsal, Reilly, and Paltsev (2012) estimated a significant welfare cost of China’s air pollution [49], and Yang and Zhang (2018) associated air pollution with significant increases in healthcare expenditure [50]. Moreover, Gao, Yuan, Liu, Xia, Huang, and Dong (2016) conducted a cost-benefit analysis for a series of air pollution control policies adopted in China, and found a benefit-cost ratio of 6.32 [51]. Some of these policies have been implemented for the Olympics as well.

### Air quality as a career incentive for local officials

Tables 4 and 5 estimate Eq (2). The first columns in both tables include only the key variables and city fixed effects. The results show that air pollution during the regulated phase was indeed tied to the CCPs’ career: If API increased by one in regulated cities, the probability of promotion decreased by 0.2% while that of demotion increased by 0.2%, compared to in non-regulated cities. Both are statistically significant. The effect on promotion doubled, and that on demotion tripled during the Games dates. After the game, API no longer exhibited significant effect on either promotion or demotion. These results confirm that air quality in regulated cities during regulated and the Games dates was more important for the career outcomes of CCPs, compared to other dates and other cities.

**Table 4:** The role of air quality in the promotion of CCP secretaries.

	CCP secretaries’ probability of promotion			
	(1)	(2)	(3)	(4)
API*reg*rdate	‒0.002***	‒0.002***	‒0.002***	‒0.003***
	(0.0008)	(0.0008)	(0.0008)	(0.0009)
API*reg*gdate	‒0.002*	‒0.002*	‒0.002*	‒0.0002
	(0.001)	(0.001)	(0.001)	(0.001)
API*reg*pdate	‒0.0009	‒0.001	‒0.001	‒0.0007
	(0.001)	(0.001)	(0.001)	(0.0004)
City	Y	Y	Y	Y
W	N	N	Y	Y
Z	N	N	N	Y
Year	N	Y	Y	Y
R^2^	0.0440	0.0541	0.0557	0.6546
Number of Observations	48,925	48,925	48,925	41,792

NOTE.—Standard errors clustered by city reported in the parentheses.

* p<0.1; ** p<0.05

*** p<0.01.

**Table 5:** The role of air quality in the demotion of CCP secretaries.

	CCP secretaries’ probability of demotion			
	(1)	(2)	(3)	(4)
API*reg*rdate	0.002**	0.002**	0.002**	‒0.0006
	(0.0008)	(0.0008)	(0.0007)	(0.0009)
API*reg*gdate	0.004*	0.004*	0.004*	0.004
	(0.002)	(0.002)	(0.002)	(0.002)
API*reg*pdate	‒0.0009	‒0.0008	‒0.0009	‒0.0002
	(0.0009)	(0.0009)	(0.0008)	(0.0006)
City	Y	Y	Y	Y
W	N	N	Y	Y
Z	N	N	N	Y
Year	N	Y	Y	Y
R^2^	0.0345	0.0801	0.0894	0.4965
Number of Observations	48,925	48,925	48,925	41,792

NOTE.—Standard errors clustered by city reported in the parentheses.

* p<0.1

** p<0.05; *** p<0.01.

These effects keep robust after controlling for year fixed effects (columns (2)) and city economic performance (columns (3)). However, the effect on demotion and that on promotion during the Games dates wane after a rich set of politician characteristics is included in column (4), while the effect on promotion during regulated dates remains robust. This implies that the observed difference in career outcomes is partly driven by selection, with more capable CCPs appointed to essential cities.

Tables 6 and 7 explore similar effects for mayors. We find that API did not significantly affect the probability of mayors’ promotion and demotion in any city and any period; nor is there any significant difference between regulated and non-regulated cities.

**Table 6:** The role of air quality in the promotion of mayors.

	Mayors’ probability of promotion			
	(1)	(2)	(3)	(4)
API*reg*rdate	‒0.004	‒0.004	‒0.004	‒0.005
	(0.003)	(0.003)	(0.003)	(0.003)
API*reg*gdate	0.003	0.003	0.004	0.005
	(0.004)	(0.004)	(0.004)	(0.003)
API*reg*pdate	‒0.001	‒0.001	‒0.001	‒0.001
	(0.002)	(0.002)	(0.002)	(0.001)
City	Y	Y	Y	Y
W	N	N	Y	Y
Z	N	N	N	Y
Year	N	Y	Y	Y
R^2^	0.0643	0.2101	0.2419	0.5434
Number of Observations	47,958	47,958	47,958	43,980

NOTE.—Standard errors clustered by city reported in the parentheses.

* p<0.1; ** p<0.05; *** p<0.01.

**Table 7:** The role of air quality in the demotion of mayors.

	Mayors’ probability of demotion			
	(1)	(2)	(3)	(4)
API*reg*rdate	0.004	0.004	0.004	0.004
	(0.003)	(0.003)	(0.003)	(0.003)
API*reg*gdate	‒0.003	‒0.002	‒0.004	‒0.003
	(0.002)	(0.002)	(0.002)	(0.002)
API*reg*pdate	‒0.0003	‒0.00008	‒0.0007	0.0003
	(0.001)	(0.001)	(0.001)	(0.0008)
City	Y	Y	Y	Y
W	N	N	Y	Y
Z	N	N	N	Y
Year	N	Y	Y	Y
R^2^	0.0851	0.1030	0.1380	0.5008
Number of Observations	47,958	47,958	47,958	43,980

NOTE.—Standard errors clustered by city reported in the parentheses.

* p<0.1; ** p<0.05; *** p<0.01

## Robustness checks

### Effect of the two control zone policy

The two control zone policy was implemented in 1996 to reduce SO_2_ and acid rain in most polluted Chinese cities. Chen, Li and Lu (2015) found that compared to cities in non-control zones, cities in control zones reduce SO_2_ significantly after 1996 [12]. To the extent that regulated/non-regulated cities coincide with control/non-control zones and API reduces with the cut of SO_2_, our results may be driven by the two control zone policy rather than the air pollution regulations for the Olympics. To explore this, we interact control zones and different phases in Eq (1). The results, available upon request, show that cities in control zones actually saw dirtier skies during some phases, while the coefficients on regulated cities remain almost unchanged from those in Table 3. Thus, the two control zone policy could not have complicated the main results.

### Extended control group

We add cities from Zhejiang and Jiangsu provinces as additional control cities, as these two provinces are geographically near to a host city, Shanghai, but were not regulated for the Olympics. Thus, they are close enough to our definition of non-regulated cities except that they are not exactly residing in the same province as Shanghai. The results, available upon request, are again consistent with those in Tables 3–7 with same signs and minimal changes in coefficients.

### Placebo test

We move the dates of the air quality regulations and the Olympics three month earlier than their actual dates and re-run Eqs (1) and (2). For Eq (1), the coefficients on the key DD interaction terms become both small and insignificant in all specifications. For Eq (2), API in both regulated and non-regulated cities are insignificantly associated with any additional career incentives during the crucial phases, for either CCP secretaries or mayors.

## Conclusions

This paper studies the effect of mega-events on cutting air pollution in Chinese cities. Using the 2008 Olympics as an example, we find that mega-events serve as an opportunity to clear the sky. The 2008 Olympics prompted a handful of Chinese cities to fight air pollution and the effect was significant and lasting. Several months before the Olympics, when the air pollution regulations for the Game phased in, cities regulated for air pollution reduced their API by seven points compared to non-regulated cities, and by nine additional points during the game. After the game, over 60% of such effect persisted until the end of 2012.

We also look into the mechanism through which these regulations were successfully enforced at the local level. We find that the enforcement was achieved by mobilizing prefecture CCP secretaries with brighter career outlooks. The reduction of API in regulated cities during the critical phases was associated with a greater chance of promotion and a thinner chance of demotion for CCP secretaries, compared to other cities and other periods. This incentivizes CCP secretaries to comply with the regulations. We do not find similar results for mayors.

Although the blue skies during the Olympics have faded, some legacies remain. Air pollutant emission targets are now an official indicator in the evaluation of government officials, and public awareness of air pollution has been greatly increased. These will continue to benefit Chinese citizens. At the same time, as China becomes even more active in hosting mega-events, its air quality may further improve alongside these practices.

More generally, this paper shows that mega-events, aside from economic opportunities and political significance, offer an opportunity for the practice of green planning. This is a valuable opportunity for all nations, and especially for developing nations, which are more likely to encounter institutional hurdles in their journey towards greener cities.

## Supporting information

S1 Table Air pollution control measures in selected host and regulated neighboring cities. (DOCX)

S2 Table Descriptive statistics. (DOCX)
