# Supplementary material for: Beyond the ceremony: Mega-event, air quality and political career
Source: PLoS One. 2022 Feb 2;17(2):e0262470. doi: 10.1371/journal.pone.0262470 (PMC8809592; doi:10.1371/journal.pone.0262470)
Supplement: S1 Table — (DOCX) [file pone.0262470.s001.docx]

**S1 Table. Air pollution control measures in selected host and regulated neighboring cities.**

| City type | City name | Measures |
| --- | --- | --- |
| *Host cities* | Beijing | (a)-(c) (i)-(l) |
|  | Tianjin | (a)-(d) |
|  | Qinhuangdao | (a) (c) (d) (m) |
|  | Shenyang | (a) (c) (d) |
|  | Qingdao | (a)(e) (f) |
| *Neighboring cities* | Shijiazhuang | (a)-(d) |
|  | Tangshan | (b)(f) (n) (o) |
|  | Baoding | (a)-(d)(p) |
|  | Taiyuan | (a)(b)(d)(e) |
|  | Datong | (a)(c)(e) |
|  | Yangquan | (a)(c)(g) |
|  | Huhehaote | (a)(b)(g)(h) |
|  | Baotou | (a)(b)(g)(h) |
|  | Chifeng | (a)(b)(h) |
|  | Jinan | (b)(d)(f)(q) |
|  | Zibo | (a)(r)-(t) |

Notes:

(1) Measures: (a) stricter vehicle control, (b) suspend polluting plants or put on reduced schedules, (c) cut smoke emission, (d) cut dusts, (e) supervise major companies, (f) enforce environmental laws, (g) speed up desulfurization, (h) optimize power dispatching method, (i) advocate green travel, (j) halt construction, (k) road cleaning, (l) emergency management for extreme air conditions, (m) clean up polluting factories, (n) stricter management of hazardous wastes and waste chemicals, (o) evaluate environmental safety risks, (p) monitor and forecast air quality, (q) establish leadership for environmental protection, (r) accelerate the construction of key desulphurization projects, (s) forbid straw burning, (t) install cleaner facilities

(2) Hong Kong (as a host city) and its neighboring cities are not regulated for the 2008 Olympics.

(3) Shanghai (as a host city) is regulated but we are unable to obtain the data on the specific control measures.
