# Supplementary material for: Beyond the ceremony: Mega-event, air quality and political career
Source: PLoS One. 2022 Feb 2;17(2):e0262470. doi: 10.1371/journal.pone.0262470 (PMC8809592; doi:10.1371/journal.pone.0262470)
Supplement: S2 Table — (DOCX) [file pone.0262470.s002.docx]

**S2 Table. Descriptive statistics.**

| Variable | Mean | Standard deviation | Minimum | Maximum | Sample size |
| --- | --- | --- | --- | --- | --- |
| Air quality: | |  |  |  |  |
| API | 72.503 | 29.719 | 9 | 500 | 50,973 |
| Career outcome: |  |  |  |  |  |
| Promotion (yes=1) | 0.363 | 0.481 | 0 | 1 | 48,925 |
| Demotion (yes=1) | 0.160 | 0. 367 | 0 | 1 | 48,925 |
| Climate variable (**X**): |  |  |  |  |  |
| Average temperature (C^o^) | 10.693 | 11.754 | -25.233 | 35.000 | 45,911 |
| Relative humidity (100%) | 60.790 | 18.977 | 9 | 100 | 45,911 |
| Precipitation (mm) | 18.681 | 75.159 | 0 | 2881 | 42,485 |
| Average wind speed (0.1m/s) | 25.138 | 16.310 | 0 | 173 | 45,897 |
| Maximum wind speed (0.1m/s) | 56.209 | 27.084 | 11 | 288 | 45,896 |
| Maximum wind direction |  |  | Calm | NNW | 45,895 |
| Average air pressure (0.1hPa) | 9674.035 | 558.020 | 8304 | 10455 | 45,909 |
| City characteristics (**W**): |  |  |  |  |  |
| Annual GDP growth rate (100%) | 16.478 | 5.629 | 3.026 | 37.879 | 50,973 |
| Annual fiscal revenue growth rate (100%) | 22.886 | 13.733 | -42.112 | 86.480 | 50,973 |
| Politician characteristics (**Z**): |  |  |  |  |  |
| Age | 52.668 | 4.055 | 41 | 61 | 50,823 |
| Sex (female=1) | 0.972 | 0.166 | 0 | 1 | 50,973 |
| Education level (1=none; 2=primary school; 3=middle school; 4=high school; 5=college; 6=university; 7=master; 8=doctor; 9=other) | 3.071 | 0.758 | 1 | 4 | 50,973 |
| Minority ethnic group (yes=1) | 0.098 | 0.298 | 0 | 1 | 50,973 |
| Academic discipline is science, engineering, agriculture and medicine (yes=1) | 0.303 | 0.460 | 0 | 1 | 50,973 |
| Academic discipline is economics, management and law (yes=1) | 0.746 | 0.435 | 0 | 1 | 50,973 |
| Length in office by month | 31.089 | 18.217 | 1 | 87 | 50,973 |
| Years of work experience | 33.441 | 5.237 | 18 | 43 | 50,973 |
| Years of experience as a CCP member | 30.247 | 5.173 | 19 | 41 | 49,169 |
| Local (yes=1) | 0.028 | 0.166 | 0 | 1 | 50,973 |
| Worked or studied abroad (yes=1) | 0.043 | 0.202 | 0 | 1 | 50,973 |
| Worked in the central government (yes=1) | 0.071 | 0.257 | 0 | 1 | 50,973 |
| Worked in the provincial government (yes=1) | 0.773 | 0.419 | 0 | 1 | 50,973 |
| Worked in universities or research institutions (yes=1) | 0.148 | 0.355 | 0 | 1 | 50,973 |
| Worked in state-owned enterprises (yes=1) | 0.288 | 0.453 | 0 | 1 | 50,973 |
| Worked in China Communist Youth League (yes=1) | 0.381 | 0.486 | 0 | 1 | 50,973 |
| Worked in the organizational department (yes=1) | 0.239 | 0.427 | 0 | 1 | 50,973 |
| Worked in the economic sector (yes=1) | 0.050 | 0.217 | 0 | 1 | 50,973 |
| Worked as a secretary (yes=1) | 0.393 | 0.489 | 0 | 1 | 50,973 |
| Social network index | 0.058 | 0.073 | 0 | 0.357 | 45,494 |
